# What the fox? Cryptic *Eucoleus* [*Capillaria*] sp. in the respiratory tract of a cat from Australia

**DOI:** 10.1016/j.crpvbd.2021.100028

**Published:** 2021-07-16

**Authors:** Nichola Eliza Davies Calvani, Megan Wright, Joanna White, Ben Stepkovitch, Emily Francis, Phoebe Rivory, Bianca Wong, Thea Wilson, Madalyn Walker, Patricia Martin, Christopher Dickman, Jan Šlapeta

**Affiliations:** a Sydney School of Veterinary Science, Faculty of Science, University of Sydney, 2006, New South Wales, Australia; b Molecular Parasitology Laboratory, Centre for One Health Ryan Institute, National University of Ireland, H91 DK59, Galway, Ireland; c Bungendore Veterinary Surgery, 112b Molonglo St, Bungendore, 2621, New South Wales, Australia; d Small Animal Specialist Hospital (SASH), Level 1, 1 Richardson Place, North Ryde, 2113, New South Wales, Australia; e Hawkesbury Institute for the Environment, Western Sydney University, Richmond, NSW, 2753, Australia; f School of Life and Environmental Sciences, Faculty of Science, University of Sydney, 2006, New South Wales, Australia

**Keywords:** *Aelurostrongylus abstrusus*, *Capillaria aerophila*, *Eucoleus aerophilus*, Feline lungworm, Mitochondrial genome, Next-generation sequencing

## Abstract

*Eucoleus aerophilus* (syn. *Capillaria aerophila*) is a zoonotic trichuroid nematode parasite of dogs, cats and wild carnivores with a global distribution. The main reservoir species in Europe is the red fox, where it has been detected in up to 97% of animals surveyed. Despite the burgeoning feral cat and fox population in Australia, there is a paucity of information about the occurrence and molecular identity of *E. aerophilus* in these species. The occurrence of a gravid capillariid nematode in the bronchoalveolar lavage of a 12-week-old kitten from central New South Wales (NSW), with a history of lower respiratory signs that had been non-responsive to treatment with metronidazole or amoxicillin-clavulanic acid, prompted a detailed morphological and molecular investigation into the identity of the parasite including the examination of opportunistically-collected red fox tracheas from the region. A combination of PCR and next-generation sequencing yielded the first complete mitochondrial genome of *E. aerophilus*, collected from the red foxes in Australia, and revealed the presence of a cryptic *Eucoleus* [*Capillaria*] sp. in the kitten from central NSW. The protein-coding genes were 14–23% and 5–30% different (pairwise distance) at the nucleotide and amino acid sequences, respectively, which suggests the occurrence of a genetically distinct *Eucoleus* sp. lineage in Australia. The phylogenetic analysis using both Bayesian and the maximum likelihood methods demonstrated monophyly of the Trichuridae plus Capillariidae using amino acid sequences encoded by mitochondrial DNA. Analysis based on complete *SSU* rDNA sequences of *Eucoleus* [*Capillaria*] sp. and *E. aerophilus* placed them within *Eucoleus* spp. from the respiratory tract of their hosts. While *Eucoleus* spp. may not currently pose a significant threat to companion animals in Australia, their status as a recently emerged pathogen in Europe suggest that greater efforts should be made to understand the distribution and epidemiology of these parasites.

## Highlights

• Discovery of a cryptic *Eucoleus* [*Capillaria*] sp. in the respiratory tract of a kitten.
• Morphological and molecular comparison of *Eucoleus aerophilus* from red foxes with the cryptic *Eucoleus* [*Capillaria*] sp.
• First two complete mitochondrial genomes for the Capillariidae assembled using whole genome next-generation sequencing.

## Graphical abstract

**Figure:**
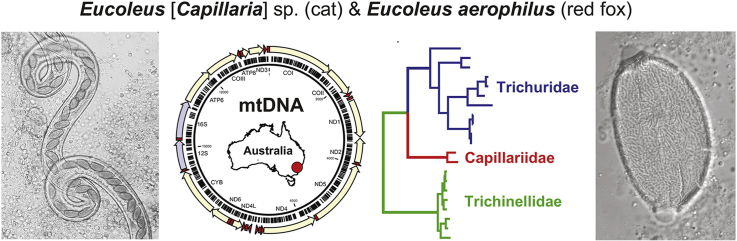

## 1 Introduction

*Eucoleus aerophilus* (syn. *Capillaria aerophila*) (Trichocephalida: Capillariidae) is a trichuroid nematode parasite of the upper respiratory tract of dogs, cats, wild carnivores and occasionally humans (Lalosevic et al., 2008; Di Cesare et al., 2012). The parasite has a direct life-cycle in which adult worms embed themselves in the mucosa of the trachea, bronchi and bronchioles where they subsequently reproduce. Eggs are coughed up in sputum, swallowed by the host and shed in faeces, where they embryonate and remain viable for up to one year (Traversa et al., 2011). Infection occurs when uninfected individuals either ingest the embryonated eggs directly or through the ingestion of infected earthworms acting as paratenic hosts (Di Cesare et al., 2012; Giannelli et al., 2017). Upon hatching in the intestines, the larvae migrate *via* the bloodstream to the upper respiratory tract. Infection with *E. aerophilus* is commonly asymptomatic, with young animals infected with a heavy burden or co-infected with other pathogens, usually those resulting in immunosuppression – such as Feline Immunodeficiency Virus (FIV) and Addison’s disease – most likely to display clinical signs (Barrs et al., 2000; Burgess et al., 2008; Elhamiani Khatat et al., 2016; Crisi et al., 2017; Morelli et al., 2021a). Clinical signs are non-specific and include generalized respiratory distress, wet to dry coughing, sneezing, wheezing and nasal discharge accompanied by lethargy, fever, depression and weight loss (Traversa et al, 2009a, 2010; Crisi et al., 2017). Occasional zoonotic transmission has been reported in regions of high prevalence (Lalosevic et al., 2008, 2013).

The epidemiology of *E. aerophilus* is largely unknown, yet its distribution is widespread, with red foxes (*Vulpes vulpes*) regarded as the main reservoir species (Nevárez et al., 2005; Lalosevic et al., 2013). High prevalence of infection in red fox populations has been observed across the northern hemisphere in helminth surveys, particularly in Europe, where estimates range from 39% in Great Britain to 97% in Lithuania (Morgan et al., 2008; Bruzinskaite-Schmidhalter et al., 2012). In domestic cats (*Felis catus*) in Europe *E. aerophilus* is considered as having recently emerged as a respiratory pathogen of concern, with a reported prevalence of 3.1% and 5.5% in Romania and Italy, respectively (Traversa et al., 2009a; Mircean et al., 2010). Reasons for this emergence have been speculated, with the impacts of global warming, urbanisation and subsequent changes in the dynamics of local wildlife populations the likely basis for the high transmission between red foxes and domestic cats (Traversa et al., 2009a, 2010).

A lack of current prevalence data makes it difficult to determine the importance of *E. aerophilus* in Australian carnivores. Previous reports from post-mortem analysis of rescue and feral cats from New South Wales and Tasmania suggest that the prevalence of *E. aerophilus* in domestic cats is low at 3 and 5%, respectively (Holmes & Kelly, 1973; Milstein & Goldsmid, 1997). A retrospective analysis of feline bronchoalveolar lavage (BAL) samples submitted for diagnosis to the University of Sydney Veterinary Centre (now the University of Sydney Veterinary Teaching Hospital) between 1995 and 2000 reported a single case of *E. aerophilus* infection in a cohort of 80 cats presenting with non-specific signs of respiratory disease (Foster et al., 2004). Red foxes are the reservoir hosts of *E. aerophilus* in Europe and are considered an invasive pest species in Australia, with densities up to 16 animals/km^2^ in urban centres (Marks & Bloomfield, 1999). Despite occurring at such high densities, the prevalence of *E. aerophilus* in Australian red foxes remains unknown.

The diagnosis of *E. aerophilus* in domestic carnivores traditionally occurs during faecal flotation or direct smear and subsequent morphological identification of eggs (Traversa et al., 2010). Diagnosis may also occur *via* microscopic examination of BAL samples, a more invasive option, which is less frequently employed (Foster et al., 2004). Due to morphological similarities with other trichuroid eggs, such as those of *Trichuris vulpis* and *Eucoleus boehmi* (syn. *Capillaria boehmi*), misdiagnosis may occur in infected canids (Traversa et al, 2010, 2011; Morelli et al., 2021b). PCR-based methods have been developed to increase diagnostic specificity but are not currently commercially available (Di Cesare et al., 2012).

The aim of this study was to determine the identity of trichuroid eggs recovered from a kitten with non-specific respiratory signs from rural New South Wales (NSW), Australia. To do so we conducted detailed morphological analysis of eggs collected from BAL in the absence of complete adult specimens. We used PCR and whole genome next-generation sequencing (NGS) to determine the complete mtDNA sequence. The material was compared to the complete mtDNA of adult *E. aerophilus* collected from feral Australian red foxes. The newly obtained mtDNA represents the first complete mtDNA sequences for the family Capillariidae Railliet, 1915 and provides evidence of a genetically distinct lineage of *E. aerophilus* in Australia.

## 2 Materials and methods

### 2.1 Capillariid from a domestic cat

A 12-week-old male rescue kitten (Domestic Medium Hair) from Orange (central western NSW, Australia) with a 6–8-week history of lower respiratory signs and no response to metronidazole or amoxicillin-clavulanic acid was presented to the Small Animal Specialist Hospital in Sydney, New South Wales, Australia in mid-January, 2018. The kitten was clinically stable but continued to have a moist cough. Radiographs showed a severe bronchointerstitial pattern with a possible small volume of pleural fluid. A faecal sample and BAL were submitted to Veterinary Pathology Diagnostic Services (VPDS), The Sydney School of Veterinary Science, at The University of Sydney, for routine diagnosis by a registered veterinarian as part of their diagnostic workup. All samples have been de-identified for the purpose of this report. A routine faecal examination, Baermann and faecal flotation with saturated NaCl (specific gravity 1.21), were conducted according to standard operational procedures alongside direct examination of the BAL. First-stage (L1) larvae of *Aelurostrongylus abstrusus* and capillariid eggs were examined under a light microscope equipped for Nomarski interference contrast (Olympus BX60), while an individual adult capillariid worm was removed from the BAL specimen and stored in 70% ethanol prior to molecular analysis.

### 2.2 Feral foxes

Twenty frozen tracheas collected from euthanised feral foxes in NSW were provided to The University of Sydney’s Veterinary Parasitology Diagnostic Laboratory as part of a research project being undertaken at Western Sydney University (Stepkovitch et al., 2019). Each trachea was slow thawed overnight and flushed with approximately 15 ml of 0.9% phosphate-buffered saline (PBS; pH = 7.4). The tracheas were dissected longitudinally and examined for adult worms with a magnifying glass. Mucosal scrapings were collected, and visible worms were extracted *via* rinsing with PBS. A new sterile scalpel blade was used for each trachea and benches were washed down between samples to avoid cross-contamination. Each scraping sample was examined under a stereomicroscope (Olympus SZH) for the presence of eggs or adult worms. Any eggs or worms suspected to be *Eucoleus* sp. were transferred to individual glass slides, moistened with PBS and viewed under an Olympus BX60 light microscope equipped for Nomarski interference contrast for identification according to morphological features described by Traversa et al. (2011). Individual adult worms were stored in 70% ethanol prior to molecular analysis.

Tracheal fluid (∼8 ml) from each specimen was centrifuged in a test tube at 1500 rpm (300× *g*) for 6 min (Spintron GT-10S). The supernatant was aspirated and approximately 12 ml of saturated NaCl solution was added to the pellet and homogenised, with a further 3 ml added until a meniscus formed. A coverslip was placed on top and allowed to stand for 10 min. The coverslip was then examined under a light microscope (Olympus BX41, Australia) at 100× magnification for the presence of eggs.

### 2.3 Molecular analysis

Total genomic DNA was isolated from a single female adult worm collected from the BAL of the kitten (Orange), from four individual worms collected from fox #F160 (#F160-1 to #F160-4), and from a further single worm collected from fox #F159 using the Isolate II Genomic DNA kit (BioLine, Australia) according to the manufacturer’s instructions, eluted in a final volume of 100 μl and stored at −20 °C until analysis.

A set of primers amplifying nematodes, Cox1NEMF [S0823] (5′-CCT GAG GTT TAT ATT YTW RTT-3′) and Cox1NEMR [S0824] (5′-CCT GTT ARR CCT CCR ATA CT-3′) were used to amplify a 344-bp sequence of the cytochrome *c* oxidase subunit 1 gene (*cox*1) (Di Cesare et al., 2012). Conventional PCR reactions were run at a total volume of 30 μl including 2 μl of DNA, 15 μl of MyTaq Red Mix (BioLine, Australia) and primers at a final concentration of 250 nM each. Reactions were performed in a Veriti 96 Well Thermal Cycler (Applied Biosystems) according to the following protocol: initial denaturation at 95 °C for 1 min, followed by 35 cycles of 95 °C for 15 s, 48 °C for 15 s, and 72 °C for 10 s. A SYBR-chemistry qPCR was performed on the individual worm DNA samples using the same primers at a final volume of 20 μl, containing 2 μl of DNA, 10 μl of SensiFAST SYBR No-ROX mastermix (BioLine, Australia) and primers at a final concentration of 100 nM. Reactions were performed in a BioRad CFX96 Real-Time Touch System with initial denaturation occurring at 95 °C for 3 min, followed by 40 cycles of 95 °C for 5 s, 50 °C for 10 s and 72 °C for 10 s, before a standard melt curve analysis.

Partial *cox*1 was amplified using primers LCO1490 and HCO2198 *via* conventional PCR (Folmer et al., 1994). PCR mixtures were prepared as described above and the reaction was run as follows: initial denaturation at 95 °C for 1 min; 35 cycles of 15 s at 95 °C, 15 s at 55 °C and 10 s at 72 °C; and a final elongation at 72 °C for 7 min. A no-template control was included with each PCR amplification. Amplicons were visualised on a 1.5% agarose gel stained with 0.1% GelRed Nucleic Acid stain (Biotium, USA). PCR products of the expected size were bi-directionally sequenced using their respective amplification primers at Macrogen Ltd. (Seoul, South Korea). Chromatograms were assembled using CLC Main Workbench 21 (CLC bio, Qiagen, Australia).

### 2.4 Sequencing and assembly of *Eucoleus* [*Capillaria*] sp. mtDNA from the whole genome sequencing data

DNA isolated from the adult worm collected from the kitten (Orange; JS4271) and from a worm collected from fox #F160 (#F160-1; JS4640) were used for Illumina Nextera XT library construction followed by next-generation sequencing (NGS) using 100 bp paired end Illumina HiSeq 2500 sequencing systems at a depth of 1 Gb of raw sequence data (Macrogen, Seoul, Republic of Korea). The complete mitochondrial genome (mtDNA) was assembled from FastQ data using the MITObim pipeline (Hahn et al., 2013) [https://github.com/chrishah/MITObim] with the aid of the DNA sequence obtained using LCO1490/HCO2198 primer pair amplicon as bait. The assembly was repeated three times with varying percentages of the raw FastQ sequence data (10–100%), keeping mtDNA coverage at 60–100×. The obtained mtDNA was annotated with the aid of MITOS Web Server (Bernt et al., 2013) [http://mitos.bioinf.uni-leipzig.de/] and aligned with available *Trichuris* spp. and *Trichinella* spp. complete mtDNA sequences in CLC Genomics Workbench 21 for manual validation.

### 2.5 Phylogenetic analysis of the order Trichocephalida based on complete mtDNA sequences

Multiple sequence alignments of all available *Trichinella* spp. and *Trichuris* spp. codon aligned sequences were constructed, and outgroup sequences from related nematodes were selected. The alignment, translation to amino acids and extraction of amino acid sequences from 12 protein-coding regions was performed with the aid of CLC Genomics Workbench 21. The amino acid alignment included 33 taxa and 2,991 amino acid residues. Phylogenetic trees were constructed using maximum likelihood and Bayesian methods. The Bayesian trees were reconstructed using MrBayes 3.2.7 (Ronquist et al., 2012). The MrBayes parameter was set to the rate matrix for amino acid data with averaging model as ‘prset aamodelpr ​= ​mixed’. A proportion of sites were set to be invariable (+I), while the rate for the remaining sites were drawn from a gamma distribution (+G) as ‘lset rates ​= ​invgamma’. The Markov chain was run for 20,000,000 generations and sampled every 200 generations. Posterior probabilities were calculated from trees from the stationary phase by discarding 25% of the initial samples. The stationary phase was evaluated using Tracer 1.7.1 (Rambaut et al., 2018) [http://beast.community/tracer]. Maximum likelihood trees were reconstructed using the Maximum Likelihood and General Reversible Mitochondrial (GTR) ​+ ​Freq. model using selection model function in MEGA X v.10.1.8 (Kumar et al., 2018) [https://www.megasoftware.net/] and the percentage of trees (bootstrap support) in which the associated taxa clustered together was calculated.

### 2.6 Assembly of *SSU* rDNA sequences and phylogenetic analysis of the family Capillariidae

The complete *SSU* rDNA was assembled from FastQ data using the MITObim pipeline (Hahn et al., 2013) with the aid of *SSU* rDNA from *E. aerophilus* (accession number MF599385) as bait. The assembly was repeated three times with varying percentages of the raw FastQ sequence data used (2–20%), keeping mtDNA coverage at < 100×. We retrieved all (*n* = 93) available *SSU* rDNA sequences belonging to the family Capillariidae from GenBank, as well as four *T. vulpis* sequences as an outgroup. New sequences obtained in this study were aligned to the initial 99 sequence data set and manually adjusted by eye using CLC Main Workbench 21. Inspection of the alignment and reduction of the redundant and short sequences formed a final alignment of 29 sequences (2,006 nucleotide residues). Phylogenetic trees were constructed using maximum likelihood and Bayesian methods. MrBayes model parameters were set for GTR ​+ ​G ​+ ​I model ‘lset nst ​= ​6 rates ​= ​invgamma; prset ratepr ​= ​variable’. The Markov chain was run for 5,000,000 generations and sampled every 200 generations. Posterior probabilities were calculated from trees from the stationary phase by discarding 25% of the initial samples with the aid of Tracer. Maximum likelihood trees were reconstructed using Maximum Likelihood method and Tamura 3-parameter model with G ​+ ​I based on selection model function in MEGA X and the percentage of trees (bootstrap support) in which the associated taxa clustered together was calculated.

## 3 Results

### 3.1 Coinfection of *Eucoleus* [*Capillaria*] sp. and *A. abstrusus* in a kitten from rural NSW

**Fig. 1:**
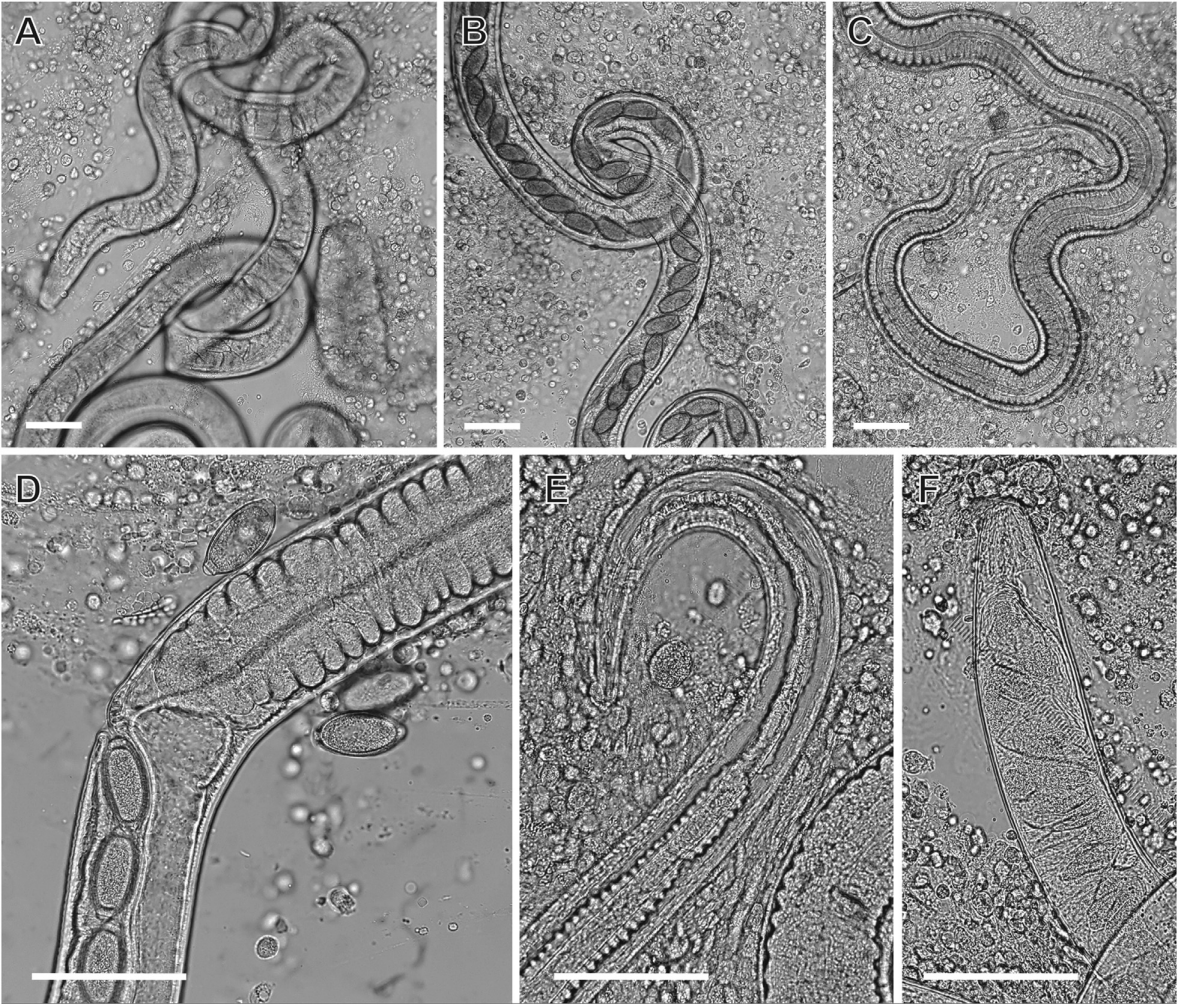
Adult female *Eucoleus* [*Capillaria*] sp. nematode from a cat from central New South Wales, Australia. Posterior extremity of the specimen (**A**), uterus of the specimen with eggs (**B**) and the anterior extremity (**C**). Magnified area of the vulva with adjacent freed eggs (**D**). Magnified anterior (**E**) and posterior end (**F**). The specimen was obtained from the bronchoalveolar lavage (BAL) of a 12-week-old kitten and was observed fresh and unpreserved. *Scale-bars*: 100 μm

**Fig. 2:**
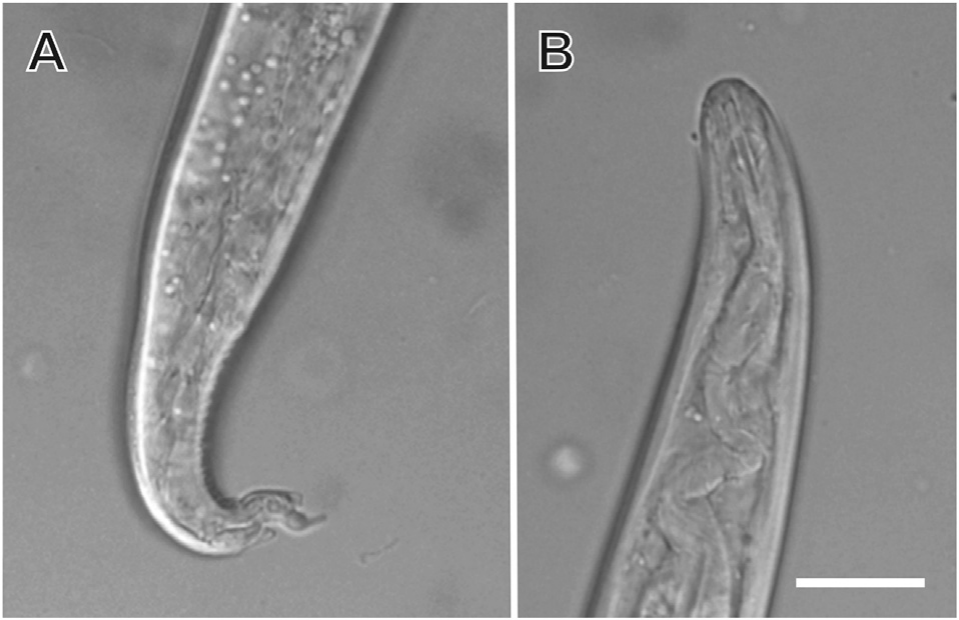
*Aelurostrongylus abstrusus* first-stage (L1) larva. **A** Posterior extremity. **B** Anterior extremity. The specimen was obtained from the bronchoalveolar lavage (BAL) of a 12-week-old kitten and was observed fresh and unpreserved. *Scale-bar*: 10 μm

**Fig. 3:**
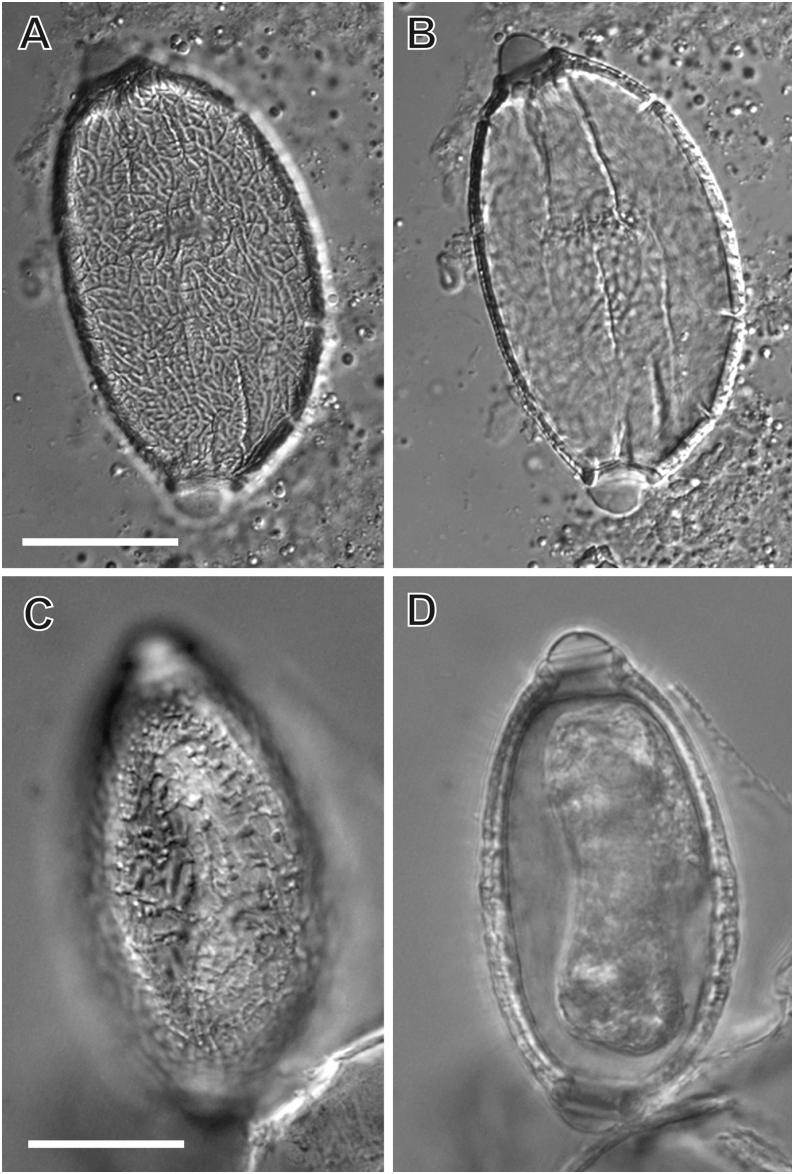
Nomarski interference contrast of the eggs of *Eucoleus* [*Capillaria*] sp. (cat) and *Eucoleus aerophilus* (syn. *Capillaria aerophila*). Surface of *Eucoleus* sp. (cat) egg demonstrating fine regular anastomosing reticulations (**A**) when focused on the surface; when focused on the asymmetrical polar plugs (**B**) the reticulation is not recognisable. Surface of *E. aerophilus* (syn. *C. aerophila*) egg demonstrating coarse anastomosing ridges (**C**) when focused on the surface; when focused on the asymmetrical polar plugs (**D**) the reticulation is not recognisable. *Scale-bars*: 20 μm

Examination of a BAL sample collected from a 12-week-old kitten suffering non-specific respiratory signs revealed a large number of motile L1 larvae of *A. abstrusus* and a single live adult female *Eucoleus* [*Capillaria*] sp. nematode (Fig. 1, Fig. 2). The female *Eucoleus* [*Capillaria*] sp. worm laid eggs during microscopic examination that were further present in low numbers in the BAL (Video S1). Faecal examination after Baermann testing revealed a large number of motile *A. abstrusus* L1 and faecal flotation in saturated NaCl revealed low numbers of *Eucoleus* sp. eggs. Detailed morphological examination of the eggs under Nomarski interference contrast suggested the presence of *Eucoleus* [*Capillaria*] sp., with asymmetric bipolar plugs and the ornamentation of the eggs composed of fine regular reticulations resembling but not matching that of *E. aerophilus* (syn. *C. aerophila*) (Fig. 3A and B). Average (± standard deviation, SD) egg width was 35.2 ± 1.10 μm, and length was 65.1 ± 1.63 μm (*n* = 10).

Supplementary video related to this article can be found at https://doi.org/10.1016/j.crpvbd.2021.100028.

The following is the supplementary data related to this article:

**Video S1** Female *Eucoleus [Capillaria]* sp. worm laying an egg during microscopic examination of the BAL collected from a 12-week-old kitten suffering non-specific respiratory signs. Video S1

The kitten was administered two doses of selamectin (Revolution for kittens, Zoetis, Australia; 15 mg, cat weight 1–1.5 kg) three weeks apart. Respiratory signs partially improved during and post treatment. In mid-March 2018 (4 weeks post-treatment) a faecal examination was repeated and revealed moderate numbers of motile *A. abstrusus* L1 using a Baermann’s test. No *Eucoleus* sp. eggs were observed during the subsequent faecal flotation. The kitten was administered two doses of emodepside (Profender for small cats & kittens, Bayer, Australia; 21.4 g/l emodepside 85.8 g/l praziquantel, cat weight 0.5–2.5 kg) two weeks apart. Respiratory signs resolved completely and parasitological examinations in mid-April 2018 were negative for both *A. abstrusus* larvae and *Eucoleus* [*Capillaria*] sp. eggs.

**Fig. 4:**
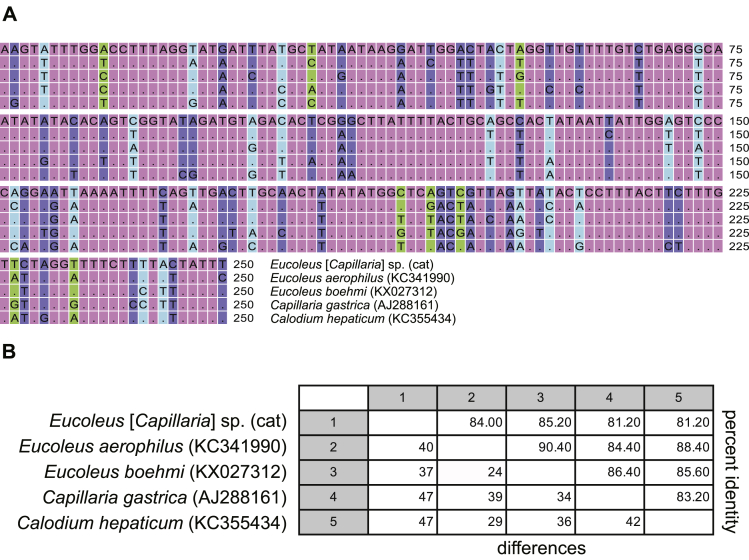
Comparison of partial nucleotide sequence of the hypervariable region of cytochrome *c* oxidase subunit 1 (*cox*1). **A** Multiple sequence alignment of partial hypervariable *cox*1 region (**A**) with related sequences identified by species name and accession GenBank number. Only the fully overlapping region is shown (250 nt). Identical residues are depicted as dots. **B** Pairwise comparison of *Eucoleus* [*Capillaria*] sp. (cat) *cox*1 with related *Capillaria* spp. using both number of differences and percent identity. The calculation was based on the alignment region shown in **A**

DNA extracted from the single adult *Eucoleus* [*Capillaria*] sp. worm collected from the kitten (Orange) and amplified using *cox*1 PCR primers (Cox1NEMF [S0823] and Cox1NEMR [S0824]) revealed a PCR product of expected size (∼350 bp; accession number MH665362) by both conventional and real-time PCR. The sequenced DNA amplicons were only 81–85% identical to capillariid nematode gene sequences from *E. aerophilus* (syn. *C. aerophila*), *Eucoleus boehmi*, *Capillaria gastrica* and *Capillaria hepatica cox*1 in GenBank (KC341990, KX027312, AJ288161, KC355434) (Fig. 4). We successfully amplified the larger *cox*1 PCR product (∼650 bp; accession number MH665361), but there were no sequences of *E. aerophilus* (syn. *C. aerophila*) or other capillariid species available for comparison.

### 3.2 Red foxes infected with *E. aerophilus* (syn. *C. aerophila*) in Australia

Two fox trachea samples were positive for *Eucoleus* [*Capillaria*] sp. worms upon visual inspection during post-mortem examination (*n* = 20; 10%; 95% CI: 3–30%). A single worm was retrieved from one fox (#F159), while a second fox (#F160) presented with a heavy infestation (>20) of small, slender white worms (∼30 mm long) and *Eucoleus* [*Capillaria*] sp. eggs that readily detached from the tracheal mucosa when flushed. Closer visual inspection confirmed both the worms and eggs as *E. aerophilus* (syn. *C. aerophila*) based on the observation of morphological characteristics specific to this genus and species, including the asymmetric bipolar plugs and coarse anastomosing ridges on the surface of the eggs; with stichosomes present in the adult worms (Moravec, 2000; Spratt, 2006; Traversa et al., 2011). The average egg width was 33.4 ± 1.06 μm, and length was 65.5 ± 1.57 μm (*n* = 10) (Fig. 3C and D).

DNA extracted from five adult worms collected from the two foxes (#F159, #F160-1 to #F160-4) was amplified using *cox*1 PCR primers (Cox1NEMF [S0823] and Cox1NEMR [S0824]) and produced products of expected size (∼350 bp; accession numbers MW709563-MW709567) using both conventional and real-time PCR. The sequenced DNA amplicons were 100% identical to each other and 100% identical to *E. aerophilus* (syn. *C. aerophila*) *cox*1 gene sequences in GenBank (JQ905052-JQ905059). We then successfully amplified the larger *cox*1 PCR product (∼650 bp; accession number MW709568). The percent identity between the newly obtained Australian *cox*1 DNA sequences; *Eucoleus* [*Capillaria*] sp. (cat) and *E. aerophilus* (syn. *C. aerophila*) (red foxes #F159 and #F160-1 to #F160-4)*,* was 86% (14% distance, 94/667 nt).

### 3.3 Complete mitochondrial genomes of *Eucoleus* [*Capillaria*] sp. and *E. aerophilus* (syn. *C. aerophila*)

**Fig. 5:**
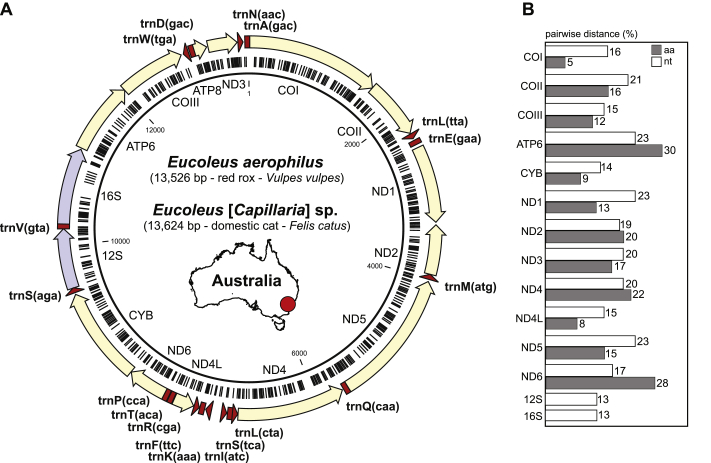
Complete mitochondrial genomes of *Eucoleus* [*Capillaria*] sp. (cat) and *Eucoleus aerophilus* (syn. *Capillaria aerophila*). **A** Diagram of mitochondrial genomes (mtDNA) of *Eucoleus* [*Capillaria*] sp. obtained from a single specimen from a cat; length indicated on the annotation and protein-coding gene regions, rRNA gene and tRNA regions are annotated. Dashes on the inner circle localize SNP sites from *E. aerophilus* (syn. *C. aerophila*) (#F160-1) from a red fox. Map indicates the approximate collection site in New South Wales, Australia. **B** The graph shows the highest pairwise nucleotide and amino acid sequence distances between the two mtDNA genomes

To better understand the molecular identity of the cat *Eucoleus* [*Capillaria*] sp. isolate and the *E. aerophilus* (syn. *C. aerophila*) collected from one of the red foxes (#F160-1) we used the isolated DNA for low coverage whole genome NGS. Using MitoBIM, we assembled the mtDNA for both specimens. Three independent MitoBIM runs returned identical mtDNA sequences. With the aid of the MITOS Web Server and subsequent manual correction, 13 protein-coding genes, 22 trn regions and two ribosomal subunits were annotated (Fig. 5A). The partial *cox*1 sequence obtained by PCR (primers Cox1NEMF-Cox1NEMR, *c.*350 bp and LCO1490-HCO2160, *c.*650 bp) from the same initial DNA following Sanger sequencing was 100% identical to the mtDNA assembled from the NGS data. The complete mitochondrial genome of *Eucoleus* [*Capillaria*] sp. (cat) was 13,624 bp (MH665363) and *E. aerophilus* (syn. *C. aerophila*) from the red fox (#F160-1) was 13,526 bp (MW722546) (Fig. 5A). The protein-coding genes were 14–23% and 5–30% different (pairwise distance) at the nucleotide and amino acid sequences, respectively (Fig. 5B).

### 3.4 Phylogeny of the order Trichocephalida based on complete mtDNA coding genes

**Fig. 6:**
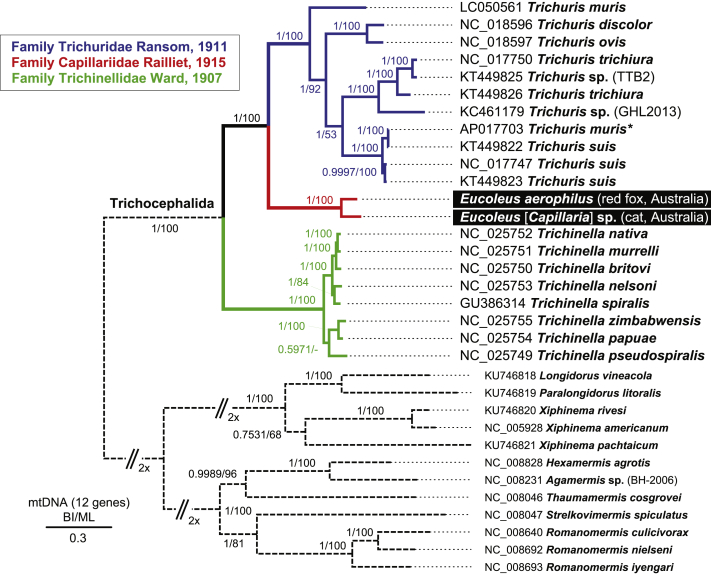
Phylogeny of Trichocephalida based on complete mitochondrial sequences. The order Trichocephalida is represented by three robust highly resolved clades representing three families: Trichuridae (blue); Capillariidae (red); and Trichinellidae (green). The evolutionary history was inferred by using Bayesian inference (BI) and maximum likelihood (ML). The amino acid sequence alignment of 2,991 amino acid residues includes concatenated 12 coding genes from available complete mtDNA genomes (2 newly obtained *Eucoleus* taxa, 11 *Trichuris* taxa, 8 *Trichinella* taxa and 12 outgroup taxa). The Bayesian tree was reconstructed using MrBayes 3.2.7 with parameter set to the rate matrix for amino acid data with averaging model with invariable (+I) sites and a gamma distribution (+G). Maximum likelihood trees were reconstructed using the maximum likelihood and general reversible mitochondrial (GTR) ​+ ​Freq. model using selection model function in MEGA X v.10.1.8. The tree is drawn to scale. Posterior probabilities from BI/bootstrap support for ML are shown above branches. Note that AP017703 is annotated as *Trichuris muris* (∗), but likely represents misidentified *Trichuris suis*

Phylogenetic analyses using amino acid sequences from 12 coding genes (alignment of 2,991 amino acid residues) from available complete mtDNA genomes included two newly obtained *Eucoleus* taxa, eleven *Trichuris* taxa, eight *Trichinella* taxa and twelve outgroup taxa. The order Trichocephalida formed three robustly resolved clades representing three families, Trichuridae, Capillariidae and Trichinellidae (Fig. 6). The phylogenetic analysis using both Bayesian inference and the maximum likelihood method demonstrated monophyly of the Trichuridae plus Capillariidae (posterior probability 1 and bootstrap support 100%). Trichinellidae formed a sister group to Trichuridae and Capillariidae (Fig. 6).

### 3.5 Phylogeny of capillariid species based on *SSU* rDNA

**Fig. 7:**
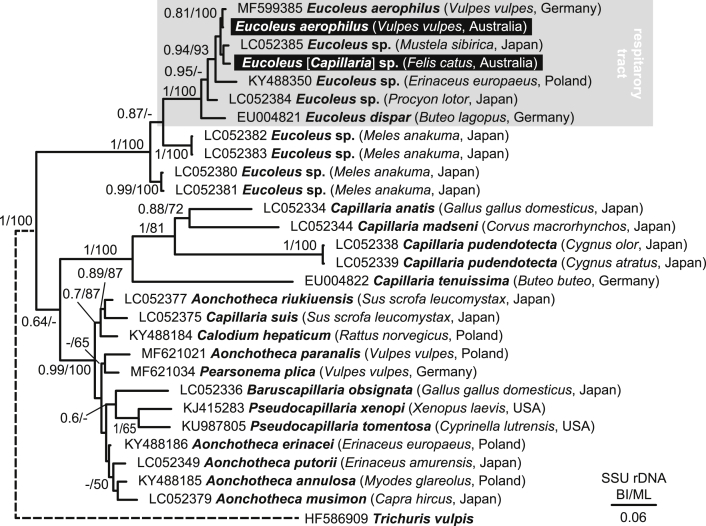
Phylogeny of the Capillariidae based on *SSU* rDNA sequences. The area of tree that includes sequences from nematodes that were removed from the respiratory tract are in grey, including *Eucoleus* [*Capillaria*] sp. from the cat and *Eucoleus aerophilus* (syn. *C. aerophila*) from a red fox obtained for this study. The evolutionary history was inferred by using Bayesian inference (BI) and maximum likelihood (ML). The nucleotide sequence alignment of 2,006 residues includes selection of Capillariidae *SSU* rDNA sequences and *T. vulpis* as an outgroup. The Bayesian tree was reconstructed using MrBayes 3.2.7 and model parameters were set for GTR ​+ ​G ​+ ​I model. Maximum likelihood trees were reconstructed using Tamura 3-parameter model with G ​+ ​I in MEGA X v.10.1.8. The tree is drawn to scale. Posterior probabilities from BI/bootstrap support for ML are shown above branches

There is currently a limited number of mtDNA sequences available to study species identity or to barcode capillariid species using mtDNA. We thus took advantage of available nuclear *SSU* rDNA, which represents a wider sample of capillariid species and isolates (Tamaru et al., 2015). To complement our mtDNA study, we used the NGS fastq reads to assemble complete *SSU* rDNA sequences. Using MitoBIM, the complete *SSU* rDNA was retrieved for both *Eucoleus* [*Capillaria*] sp. (cat) (1,846 nt) and *E. aerophilus* (syn. *C. aerophila*) (red fox #F160-1) (1,850 nt) (MW709573-MW709574)*.* The resulting alignment of *SSU* rDNA includes 28 sequences, including 26 previously published sequences and two sequences from the present study. A *T. vulpis SSU* rDNA sequence (Trichuridae) was included as an outgroup (accession number HF586909). The alignment had 2,006 nucleotide residues. Phylogenetic analysis confirmed that both *SSU* rDNA from *Eucoleus* [*Capillaria*] sp. (cat) and *E. aerophilus* (syn. *C. aerophila*) (red fox #F160-1) are closely related to sequences within the genus *Eucoleus* confined to the respiratory tract of their hosts (Fig. 7). The Australian *E. aerophilus* isolate’s *SSU* rDNA demonstrated close homology to *E. aerophilus* (99.8%, distance 3/1,749) from a red fox from Germany (MF599385), while the *Eucoleus* [*Capillaria*] sp. (cat) isolate’s *SSU* rDNA demonstrated close homology to *Eucoleus* sp. (99.2%, distance 15/1,814) from a Siberian weasel (*Mustela sibirica*) from Japan (LC052385). At *SSU* rDNA, the Australian *E. aerophilus* (syn. *C. aerophila*) (red fox #F160-1) and *Eucoleus* sp. (cat) were 99.1% identical with 16 nucleotide differences.

## 4 Discussion

The present study provides the first complete mt genomes for the family Capillariidae and is the first investigation into *E. aerophilus* (syn. *C. aerophila*) in red foxes in Australia, providing the baseline for future epidemiological investigations into *Eucoleus* spp. both locally and internationally. With over 300 recognised species, the family Capillariidae is the largest family within the medically and veterinary important enoplids. This large number of species has understandably given rise to controversy over their identity and taxonomy, especially when considering their overlapping host preferences, predilection sites and morphologies (Butterworth & Beverley-Burton, 1980; Moravec, 2000). The mt genomes generated in the present study complement the many that are already available for *Trichinella* spp. and *Trichuris* spp., helping to resolve their position within the enoplids, but further highlight the gaps left by the remaining trichocephalids. Phylogeny based on the mt encoded amino acid sequences herein demonstrated robust monophyly of *Eucoleus* spp. with *Trichuris* spp. Such monophyly confirms previous suggestions by taxonomists based on morphology alone, where the family Trichuridae is considered more closely related to Capillariidae than Trichinellidae based on key anatomical features such as the presence of a spicule, musculature of the oesophagus and ornamentation of the cirrus (Wright, 1972, 1974; Butterworth & Beverley-Burton, 1980).

Both the morphological and molecular *cox*1 identity of the Australian *E. aerophilus* (syn. *C. aerophila*) from the red fox (#F160-1) are in agreement with those described from Europe, where they are considered the reservoir host of *E. aerophilus* (syn. *C. aerophila*) infection in cats (Moravec, 2000; Lalosevic et al., 2013; Di Cesare et al., 2014). Our finding of *E. aerophilus* (syn. *C. aerophila*) adults in the tracheas of two foxes from NSW is the first investigation into the role of red foxes in the epidemiology of this parasite in Australia. The prevalence of *E. aerophilus* (syn. *C. aerophila*) in foxes from NSW was lower than that observed in Europe, where reports suggest that the prevalence in foxes can be as high as up to 97% (Bruzinskaite-Schmidhalter et al., 2012). Nevertheless, our results confirm the ubiquity and pervasiveness of this host-parasite relationship beyond Europe and, given the density of foxes and the burgeoning feral cat population across Australia, suggest that the Australian *E. aerophilus* (syn. *C. aerophila*) population is well-poised for similar emergence (Marks & Bloomfield, 1999; Abbott, 2008; Traversa et al, 2009a, 2010; Giannelli et al., 2017).

No records currently exist of a capillariid species other than *E. aerophilus* (syn. *C. aerophila*) within the lungs and trachea of cats and thus we initially assumed that the female worm recovered from the BAL of a 12-week-old kitten suffering non-specific respiratory signs was *E. aerophilus* (syn. *C. aerophila*)*.* Comparison of the *cox*1 DNA sequences generated by low coverage whole genome NGS, however, revealed that the percent identity between *Eucoleus* [*Capillaria*] sp. (cat) and *E. aerophilus* (syn. *C. aerophila*) was only 86% (14% distance, 94/667 nts). The partial *cox*1 sequences of *E. aerophilus* (syn. *C. aerophila*) isolated from pets and wild animals (dogs, cats, foxes, beech martens) across Europe and Canada are relatively conserved with high (96–100%) percent identity (Di Cesare et al., 2014). Thus, based on the information above the *Eucoleus* [*Capillaria*] sp. (cat) identified in the present study is genetically distinct from *E. aerophilus* (syn. *C. aerophila*) and represents an as-of-yet undescribed capillariid species from Australia.

Reports of *E. aerophilus* (syn. *C. aerophila*) infections in cats in Australia are limited, with only a single case published between 1995 and 2000 during a retrospective study of a cohort of animals presenting with non-specific signs of respiratory disease (Foster et al., 2004). Based on historical data the expected prevalence in the feral cat population in Australia is between 3 and 5% (Holmes & Kelly, 1973; Milstein & Goldsmid, 1997). In Europe *E. aerophilus* (syn. *C. aerophila*) was reported to be the third most-commonly diagnosed respiratory tract nematode of cats between 2015 and 2016 (Giannelli et al., 2017). The most frequently diagnosed cat lungworm was *A. abstrusus*, and together with *E. aerophilus* (syn. *C. aerophila*), occurred most commonly in animals aged from 6 months to 2 years (Giannelli et al., 2017). The kitten in the present study was similarly co-infected with *A. abstrusus* and demonstrates that the transmission pathways of both parasites are likely to be linked by paratenic hosts ingested by cats while hunting, including those that have recently ingested infected paratenic hosts themselves (Woods et al., 2003; Giannelli et al., 2017).

Species identification in earlier reports of *E. aerophilus* (syn. *C. aerophila*) infections in Australian cats was determined based on egg size and no prior molecular data are available. The results of the present study reveal that species identification based on detailed egg ornamentation is sufficient to differentiate *E. aerophilus* (syn. *C. aerophila*) from *Eucoleus* sp. (cat) using Nomarski interference contrast light microscopy, although it is unlikely that such highly specialised infrastructure will be available to all clinicians and diagnostic laboratories (Traversa et al., 2010). In the absence of other molecular data and more detailed morphological descriptions of eggs, we cannot confirm whether earlier infections in cats in Australia were *E. aerophilus* (syn. *C. aerophila*) or the *Eucoleus* [*Capillaria*] sp. identified in the present study (Holmes & Kelly, 1973; Milstein & Goldsmid, 1997; Foster et al., 2004). It therefore remains possible that the kitten diagnosed in the present study was an accidental host of a capillariid species from another mammal or native Australian marsupial (Spratt, 2006).

Mitochondrial *cox*1 has previously been used to characterise *Capillaria* spp. (*C. gastrica* and four undescribed *Capillaria* sp.) from a variety of tissues in Australian marsupials and rodents (Zhu et al., 2000). Despite representing a variety of undescribed capillariids from Australia, none of these existing *cox*1 (∼450 bp) sequences matched the *cox*1 *Eucoleus* sp. (cat) from the present study. Unfortunately, the short sequences generated by Zhu et al. (2000) do not allow further phylogenetic analysis, for which additional genes, preferably mtDNA, are required. Besides *cox*1, *SSU* rDNA was used herein to determine the species identity and confirm our *cox*1 results. Previously, *SSU* rDNA has been used to successfully resolve generic relationships within the Capillariidae (see Leis et al., 2016). Unfortunately, inter-species genetic distance between existing *SSU* rDNA sequences is < 1%, making *SSU* rDNA inadequate for capillariid species determination. The genetic distance between available *cox*1 sequences is > 10% and is therefore more robust for species differentiation within this taxon. Reviewing the available *SSU* rDNA sequences revealed several respiratory tract *Capillaria* spp. from Japan that are closely related to both *Eucoleus* [*Capillaria*] sp. (cat) and *E. aerophilus* (syn. *C. aerophila*) (Tamaru et al., 2015). The high conservation of *SSU* rDNA precluded further evaluation of conspecificity with our material, for which *cox*1 was more suitable. We therefore urge researchers to consider *cox*1 or suggest that they generate alternative markers *via* NGS for future studies characterizing capillariids.

Unlike for cat lungworm (*A. abstrusus*), there are currently no products registered for the treatment of capillariids in Australia. As far as we are aware, only Broadline spot-on solution for cats (Boehringer Ingelheim Animal Health) has previously been evaluated for its efficacy against *E. aerophilus* (syn. *C. aerophila*) *via* egg reduction in the faeces of infected cats (Knaus et al., 2015). Currently, however, this treatment is only registered by the European Medicines Agency (EMA) for the treatment of the urinary tract capillariid *Capillaria plica*, and not for *E. aerophilus* (syn. *C. aerophila*). In South Africa and Namibia, Broadline spot-on solution for cats is registered for the treatment of lungworm infestations (*A. abstrusus*, *Capillaria* spp.), but remains unregistered for use against *E. aerophilus* (syn. *C. aerophila*) in Australia. Revolution for kittens (selamectin, Zoetis, Australia) in Australia is not registered for treatment of *A. abstrusus* or *E. aerophilus* (syn. *C. aerophila*); however, previous reports have suggested its usefulness against these nematodes in cats (Grandi et al., 2005; Iannino et al., 2013). While the use of Revolution for kittens eliminated the capillariid eggs from faeces four weeks post treatment in the current study, the cat lungworm L1 remained present upon follow up. Application of Profender for small cats & kittens (emodepside, Bayer, Australia), a registered product for treatment of *A. abstrusus* infections, was successful in eliminating the cat lungworm L1, with no larvae detected in the faecal sample four weeks post treatment. Emodepside is known to be highly successful at eliminating nematode infections in cats, including infection with *A. abstrusus* (see Traversa et al., 2009b).

## 5 Conclusion

Feline respiratory infections with capillariid species are rarely diagnosed and thus it is evident that there is still much to be learnt about capillariids and their occurrence and diversity in carnivorous mammals and Australian marsupials. While *Eucoleus* spp. may not currently pose a significant threat to companion animals in Australia, their status as a recently emerged and occasionally zoonotic pathogen in Europe suggest that greater efforts should be made to understand the distribution and epidemiology of these parasites. This includes modifications to available diagnostics and evaluation of treatment options in order to update existing recommendations and to provide the necessary resources for case management in clinical settings.

## Funding

This research did not receive any specific grant from funding agencies in the public, commercial, or not-for-profit sectors.

## CRediT author statement

Nichola Calvani: Conceptualization; Data curation; Formal analysis; Investigation; Methodology; Project administration; Supervision; Validation; Roles/Writing - original draft; Writing - review & editing. Megan Wright: Investigation; Methodology; Resources; Writing - review & editing. Joanna White: Investigation; Methodology; Resources; Writing - review & editing. Ben Stepkovitch: Data curation; Investigation; Methodology; Resources; Writing - review & editing. Emily Francis: Investigation; Methodology; Roles/Writing - original draft; Writing - review & editing. Phoebe Rivory: Investigation; Methodology; Roles/Writing - original draft; Writing - review & editing. Bianca Wong: Investigation; Methodology; Roles/Writing - original draft; Writing - review & editing. Thea Wilson: Investigation; Methodology; Roles/Writing - original draft; Writing - review & editing. Madalyn Walker: Investigation; Methodology; Roles/Writing - original draft; Writing - review & editing. Patricia Martin: Investigation; Methodology; Writing - review & editing. Christopher Dickman: Supervision; Methodology; Resources; Writing - review & editing. Jan Šlapeta: Conceptualization; Data curation; Formal analysis; Funding acquisition; Investigation; Methodology; Project administration; Resources; Supervision; Validation; Visualization; Roles/Writing - original draft; Writing - review & editing.

## Data availability

The *E. aerophilus* specimens from fox #F160 were deposited in the Australian National Wildlife Collection, CSIRO, Canberra, Australian Capital Territory, Australia (accession numbers: W/L HC# N5700). Raw fastq sequence data was deposited at SRA NCBI BioProject: PRJNA707657. The nucleotide sequence data generated in this study was deposited in GenBank (NCBI): MH665361-MH665363, MW709563-MW709568, MW709573-MW709574 and MW722546. All sequence data, associated supplementary material and additional data are available at LabArchives (https://doi.org/10.25833/y5pv-8f69).

## Declaration of competing interests

The authors declare that they have no known competing financial interests or personal relationships that could have appeared to influence the work reported in this paper.
